# Experimental ovine toxoplasmosis: influence of the gestational stage on the clinical course, lesion development and parasite distribution

**DOI:** 10.1186/s13567-016-0327-z

**Published:** 2016-03-16

**Authors:** Pablo Castaño, Miguel Fuertes, Javier Regidor-Cerrillo, Ignacio Ferre, Miguel Fernández, M. Carmen Ferreras, Javier Moreno-Gonzalo, Camino González-Lanza, Juana Pereira-Bueno, Frank Katzer, Luis Miguel Ortega-Mora, Valentín Pérez, Julio Benavides

**Affiliations:** Departamento de Sanidad Animal, Instituto de Ganadería de Montaña (CSIC-ULE), Grulleros, 24346 León, Spain; SALUVET, Animal Health Department, Faculty of Veterinary Sciences, Complutense University of Madrid, Ciudad Universitaria s/n, 28040 Madrid, Spain; Moredun Research Institute, Pentlands Science Park, Bush Loan, Edinburgh, EH26 0PZ UK

## Abstract

The relation between gestational age and foetal death risk in ovine toxoplasmosis is already known, but the mechanisms involved are not yet clear. In order to study how the stage of gestation influences these mechanisms, pregnant sheep of the same age and genetic background were orally dosed with 50 oocysts of *Toxoplasma gondii* (M4 isolate) at days 40 (G1), 90 (G2) and 120 (G3) of gestation. In each group, four animals were culled on the second, third and fourth week post infection (pi) in order to evaluate parasite load and distribution, and lesions in target organs. Ewes from G1 showed a longer period of hyperthermia than the other groups. Abortions occurred in all groups. While in G2 they were more frequent during the acute phase of the disease, in G3 they mainly occurred after day 20 pi. After challenge, parasite and lesions in the placentas and foetuses were detected from day 19 pi in G3 while in G2 or G1 they were only detected at day 26 pi. However, after initial detection at day 19 pi, parasite burden, measured through RT-PCR, in placenta or foetus of G3 did not increase significantly and, at in the third week pi it was lower than that measured in foetal liver or placenta from G1 to G3 respectively. These results show that the period of gestation clearly influences the parasite multiplication and development of lesions in the placenta and foetus and, as a consequence, the clinical course in ovine toxoplasmosis.

**Electronic supplementary material:**

The online version of this article (doi:10.1186/s13567-016-0327-z) contains supplementary material, which is available to authorized users.

## Introduction

Ovine toxoplasmosis is an important infectious disease caused by the protozoan parasite *Toxoplasma gondii*, leading to important losses in the sheep industry worldwide due to abortions after primo-infection of pregnant sheep [1]. This disease is characterized by the presence of multifocal necrotic foci in the placenta and several organs of the foetus, mainly liver and brain, with mild infiltration of inflammatory cells [2, 3]. When abortions occur, the foetus and placenta are expelled usually 1 month or later, after infection of the ewes, although few experimental studies have reported the so-called “acute phase abortions” occurring during the second week after infection, where lesions are different to those classically described in ovine toxoplasmosis [4, 5]. In the acute phase abortions, thrombosis and infarcts could be found in the placentomes and leukomalacia, in the absence of glial reaction, is the only type of lesion found in the foetus [6].

Despite the worldwide importance of this disease, the mechanisms involved in the abortion remain unclear. Lesions in foetal tissues are not extensive and some lambs born with severely damaged placentas appear healthy. It is not clear whether the cause of abortion is a direct consequence of the multiplication of the parasite in the foetus or the placenta, or if it is caused by a deregulation of hormones or immune responses in the placenta [1, 7]. However, it seems clear that the presence of the parasite in the placenta is a key factor in its pathogenesis and that it is necessary for the abortion to occur [1]. A detailed study, based on the experimental infection in pregnant sheep at different times of gestation showed that the outcome of the infection heavily depends on the gestational age in which it occurs and that the effect of infection is more severe in early gestation [2]. At this stage, the risk of intrauterine transmission to the foetus increases during pregnancy, while the deleterious effects on the foetus are also more severe at this stage during development [2]. Although this same study showed, in two different experimental infections over consecutive years, that lesions, both placental and foetal, were more severe in ewes infected during the second term of gestation, when compared to infections in the first term; no further research into the underlying mechanisms responsible for these differences in ovine toxoplasmosis has been conducted.

There are similarities, in terms of histopathological changes and clinical outcome, between ovine toxoplasmosis and ovine neosporosis, a protozoan disease of sheep that is closely related to the former due to the close relation between the aetiologic agents [8]. Experimental infection of sheep with *Neospora caninum* has shown that there is a close relation between parasite burden and the severity of lesions identified in placenta or foetus, and how these parameters could affect the course of clinical disease [8]. The close relationship between the presence of *T. gondii* and the development of histological lesions has been shown through quantitative real time-PCR (qPCR), in sheep experimentally infected at mid gestation [9]. Whether there are differences in the quantity of parasite and the presence of lesions at different terms of gestation in ovine toxoplasmosis has not yet been investigated.

This study sets out to address how gestational age, at which maternal infection occurs, influences clinical disease, development of lesions and parasite distribution/burden in ovine toxoplasmosis. We have conducted experimental infections of pregnant sheep, with a similar genetic background, that have been managed under standardized conditions and these sheep were orally challenged at early, mid and late gestation with sporulated oocysts from the M4 isolate of *T. gondii*.

## Materials and methods

### Ethics statement

All protocols involving animals were approved by the Animal Welfare Committee of the Livestock Health and Production Institute (IGM, ULE-CSIC), León, Spain, following proceedings described in Spanish and EU legislations (Law 32/2007, R.D. 1201/2005, and Council Directive 2010/63/EU). All animals used in this study were handled in strict accordance with good clinical practices and all efforts were made to minimize suffering.

### Animals and experimental design

Pure Churra breed primiparous sheep aged 24–30 months, seronegative for *T. gondii*, *N. caninum*, border disease virus, *Coxiella burnetii* and *Chlamydophila abortus* were oestrus synchronized and mated with pure breed Churra tups for 2 days, after which the rams were removed from the ewes. Pregnancy and foetal viability were confirmed by ultrasound scanning on day 40 after mating. Thirty-six pregnant sheep were randomly distributed into three experimental groups. Twenty seven ewes were allocated into groups 1 (G1; *n* = 9), 2 (G2; *n* = 9) and 3 (G3; *n* = 9). Each of these sheep were orally dosed with 50 sporoulated oocysts of the M4 isolate of *T. gondii* [6] at 40, 90 and 120 days of gestation (dg), respectively. The nine remaining control sheep were also allocated into these groups, three ewes per group, and received 50 mL of PBS as negative control of inoculation.

The initial experimental design involved the scheduled serial culling of three challenged and one unchallenged-control animals at 12, 19 and 26 days post-inoculation (dpi).

### Clinical monitoring and collection of samples

Ewes were observed daily after inoculations throughout the experimental period. Rectal temperatures were daily recorded from day 0 until 12 dpi.

On the scheduled days for the serial cullings (12, 19 and 26), or when spontaneous abortion occurred, dams were sedated with xylazine–Rompun-(Bayer, Mannheim, Germany) and immediately euthanized by an IV overdose of embutramide and mebezonium iodide-T61-(Intervet, Salamanca, Spain).

Post-mortem examination of the ewes and foetuses was carried out immediately after euthanasia, and foetuses were immediately separated from the placenta. Collection of samples for serological, histological and molecular studies was as follows: blood samples were collected by jugular veni-puncture from the dams before euthanasia and, from umbilical cord veins or heart during necropsy from the foetuses, when the uterus was open during necropsy and the blood of the foetus was not already clotted. Blood samples were collected into Vacutainer tubes (Becton–Dickinson and Company, Plymouth, UK) without anticoagulant and allowed to clot. Serum was obtained by centrifugation and samples were stored at −80 °C until analysis. In most of the cases when abortion or stillbirth occurred, foetal blood was clotted, so thoracic fluid was sampled instead and kept at −80 °C until analysis. After necropsy, five placentomes were randomly selected, paying no attention at their location within the placenta and were transversally cut into slices of 2–3 mm thickness that were fixed in 10% formalin for histopathological examinations, and additional tissue was stored −80 °C for parasite DNA detection by PCR. Samples from foetal tissues, included brain, liver, heart and lungs were stored at −80 °C for DNA extraction and also fixed in 10% formalin for histopathology.

### Serological analyses: IFAT

Indirect fluorescent antibody test (IFAT) was used to detect specific IgG anti-*T. gondii* antibodies in foetal fluids or sera and sera from dams, adapting the technique previously described for IFAT analysis in *N. caninum* infected animals [10]. Briefly, foetal fluids/sera and sera from dams were diluted at two-fold serial dilutions in PBS starting at 1:8 and 1:200, respectively, to the end point titre. Purified *T. gondii* tachyzoites (M49 strain) from cell cultures were dispensed onto 18-spot IFAT glass slides, air dried at room temperature and fixed with acetone solution. After incubation with sera dilutions at 37 °C and washing twice with PBS, fluorescein isothiocyanate (FITC) conjugate anti-sheep IgG (Sigma-Aldrich), diluted 1:200 in Evans Blue (Sigma-Aldrich), was added to the slides and incubated at 37 °C for 30 min, washed twice with PBS, followed by washing with water and mounted with Fluoprep Solution (BioMérieux, France) for microscopy visualization. Unbroken tachyzoite membrane fluorescence at titre ≥8 for foetal fluids or sera and ≥200 for sera from dams was considered a positive reaction.

### Histopathology and lesion scoring

After fixation for 5 days, maternal and foetal brains were cut coronally, embedded in paraffin wax and processed with the rest of the samples, by standard procedures for haematoxylin and eosin (HE) staining. Conventional histological evaluation was carried out on all sections. To quantify the lesions in the foetal viscera and placenta, the number and size of necrotic foci, as well as the total area of lesion in the examined tissue were calculated through a computer-assisted morphometric analysis in HE stained sections following the same procedure as described previously [8]. In this case, both necrotic and inflammatory foci were evaluated in the placenta, brain, liver and lung of the foetus.

### DNA extraction and PCR for parasite detection and quantification in tissues

Genomic DNA was extracted from three 50–100 mg samples taken from each location: five placentomes and foetal brain, liver, heart, lung and skeletal muscle (femoral region) using the commercial Maxwell^®^ 16 Mouse Tail DNA Purification Kit, developed for the automated Maxwell^®^ 16 System (Promega, Wisconsin, USA), following the manufacturer’s recommendations. The concentration of DNA for all samples was determined by spectrophotometry and adjusted to 50–100 ng/µL.

*Toxoplasma gondii* DNA detection was carried out by an ITS-1 PCR adapted to a single tube following procedures previously described [11]. Each reaction was performed in a final volume of 25 μL with 5 μL of sample DNA. PCR was carried out using five samples from the placentomes, three samples each from foetal liver and brain and one sample each of lung and heart. Both reactions without template DNA and DNA samples from the foetal-control group (G4) were included in each round of DNA extraction and PCR as negative controls. In addition, positive PCR controls with a quantity of *T. gondii* genomic DNA equivalent to 10 and 1 tachyzoites in 100 ng of sheep DNA were also included in each batch of amplifications. Ten µL aliquots of the PCR products were visualized under UV light in 1.5% agarose/ethidium bromide gel. A reaction was determined as positive when a band of 227 bp was detected.

DNA from placental tissues and brain and liver from the foetuses that tested positive samples by nested-PCR were adjusted to 20 ng/μL and quantified using qPCR. Primer pairs for the 529-bp repeat element for *T. gondii* were used to quantify the parasite and primers for the β-actin gene were used to quantify host DNA [9]. Reactions were performed in a final volume of 20 μL using Power SYBR^®^ Green PCR Master Mix (Applied Biosystems, Foster City, CA, USA), 20 pmol of each primer and 100 ng of DNA in a ABI 7300 Real Time PCR System (Applied Biosystems). Amplification was performed by a standard protocol (10 min at 95 °C, 40 cycles at 95 °C for 15 s, and 60 °C for 1 min). The number of *T. gondii* tachyzoites was calculated by interpolating the average Ct values on two standard curves: 1) equivalent to 5 × 10^5^–5 × 10^−1^ tachyzoites with tenfold serial dilutions in a solution of ovine genomic DNA and 2) a curve of 320, 160, 80, 40, 20, 10, and 5 ng of genomic DNA for ovine DNA quantifications. Parasite number in tissue samples (parasite burden) was expressed as parasite number/mg ovine tissue. Standard curves for *T. gondii* and sheep DNA showed an average slope of −3.38 and −3.34, respectively, and a R^2^ > 0.99.

### Statistical analysis

Data of body temperature were analyzed by repeated measures analysis using the MIXED procedure of SAS (SAS Inst. Inc., Cary, NC, USA). As different control groups were used for each experimental challenged group (G1, G2 and G3 groups) three independent analyses were performed, including the statistical model of the fixed effects of group (control group vs challenged experimental group), time (day after infection) and its interaction. The random effect of animal nested to the experimental group was used as the error to test the group effect. The effects of time and time × group were tested against residual error. Comparisons among the three experimental challenged groups were also performed using the same model but including three levels within the group factor (G1, G2 and G3 groups). In all analyses, different covariance structures (compound symmetry, unstructured and autoregressive) were evaluated based on Schwarz’s Bayesian information criteria.

Differences in PCR detection of parasite DNA were evaluated using the *χ*^*2*^ or Fisher exact F-test using GraphPad Prism 5.0 software. Differences in parasite burdens and histological scoring were analysed using the non-parametric Kruskal–Wallis test followed by Dunn’s test for comparisons between groups, and the Mann–Whitney test for pairwise comparisons. Theses analyses were also performed with GraphPad Prism 5.0 software. Statistical significance for all analyses was established at *P* < 0.05.

## Results

### Clinical observations

**Figure 1:**
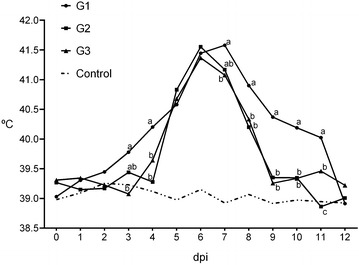
**Mean rectal temperatures of challenged and control ewes.** Mean values with different superscripts showed significant difference (*P* < 0.05).

When analysing the temperature of animals, statistically significant differences (*P* < 0.001) in the mean rectal temperature were found between challenged and control sheep (Figure 1), with variations according to group. While the mean temperature of challenged sheep from G1 was significantly higher than the control animals between days 3 and 11 pi, the period of significant differences was shorter in G2 (between days 5 and 8 pi) and G3 (between days 4 and 8 pi). When comparing between challenged groups, the mean temperature of animals from G1 was significantly higher than G2 and G3 on days 3, 4, 8, 9, 10 and 11 pi (Figure 1). Differences between G2 and G3 was only found on day 11, when it was higher in G3.

Abortions (i.e. foetal death with or without foetus expulsion from the uterus) occurred among challenged ewes in the three challenged groups (Table 1). According to time of occurrence, these abortions could be grouped into two categories: (a) abortions during the acute phase of the disease (i.e. between 9 and 14 dpi) and (b) abortions, or stillbirths occurring between 19 and 26 dpi. The occurrence of these abortions and stillbirths resulted in a modification of the number of challenged ewes culled on days 12, 19 and 26 pi for G2 and G3, as planned within the initial experimental design (Table 1).

**Table 1:** **Distribution of experimental animals according to the infection time (G1, G2 or G3) and day post infection when culled or abortion occurred**

	No of sheep					
	Abortions^a^			Serial culling		
	6–16 dpi		17–26 dpi	12 dpi	19 dpi	26 dpi
G1		2/0^b^	1/0	1/1	3/1	2/1
G2		4/0	1/0	1/1	2/1	1/1
G3		2/0	4/0	2/1	1/1	0/1

^a^Spontaneous abortions or stillbirths occurring in that group, the other time-points (12, 19 and 26 dpi) correspond to serials culling of pregnant ewes.

^b^Infected/control sheep.

**Figure 2:**
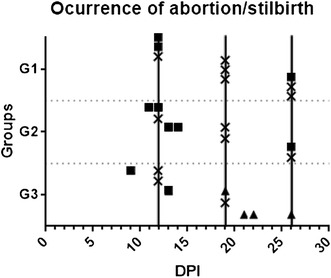
**Days post infection when foetuses where sampled.** Squares indicate abortion, triangle stillbirths and crosses showed when the foetus was studied after euthanasia of the dam at the prearranged day of serial culling (showed by a continuous vertical line).

**Figure 3:**
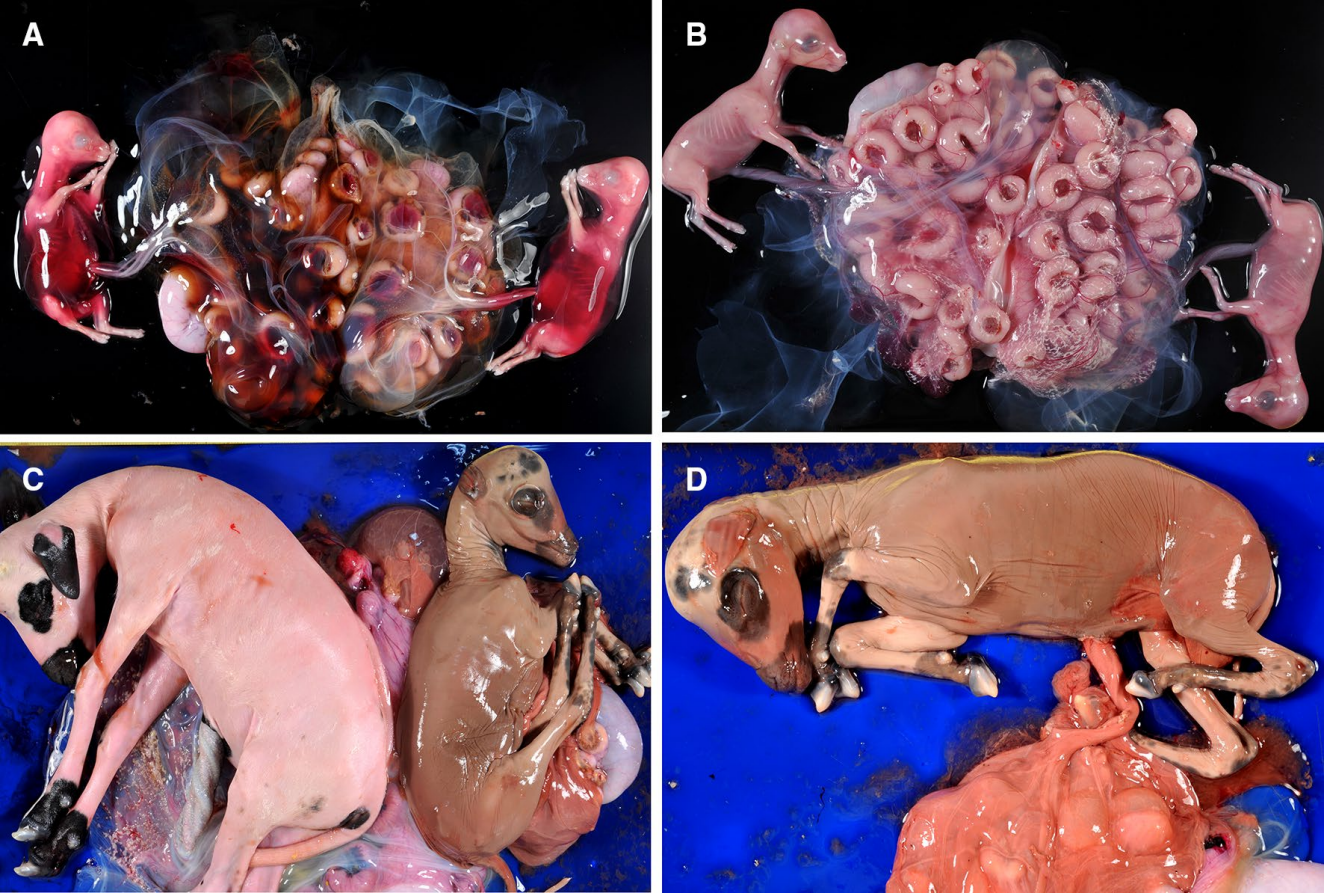
**Macroscopic lesions in the foetuses.** **A** Abdominal haemorrhage in twin foetuses from a ewe challenged at 40 dpi. Notice the congestion in the foetal part of some placentomes. **B** Twins from a control, non-infected ewe from G1. **C** Twin foetuses from a challenged ewe in G2. Foetuses showed different degree of autolysis, suggesting that their dead occurred at different days post infection. **D** Aborted foetus from G2 showing maceration and also autolysis of the placenta.

The acute phase abortions occurred as follows: in G1, two ewes culled at 12 dpi carried dead foetuses; in G2, four ewes aborted on days 11, 12, 13 and 14 pi and, in G3, two ewes aborted on days 9 and 13 pi (Figure 2). Regarding abortions that occurred after day 19 pi, one sheep from G1 and another from G2, culled both at 26 dpi, carried dead foetuses. In G3, four ewes delivered stillbirths on days 19, 21, 22 and 26 pi (Figure 2). Among the ewes from groups G2 and G3 carrying multiple foetuses (8 sheep in G2 and 4 sheep in G3) the coexistence of one smaller and mummified foetus with another viable and bigger (Figure 3) was found in three ewes, one culled at 26 dpi in G2 and two delivering stillbirths at 22 and 26 in G3.

### *Toxoplasma*-specific IgG responses

*Toxoplasma*-specific IgG responses were analysed by IFAT in foetal fluids, foetal sera and sera from dams collected immediately before necropsy. All challenged ewes were seropositive, with titres ranging from 1:400 to 1:1600 in G1, 1:400 to 1:6400 in G2 and 1:1600 to 1:12 800 in G3 (Additional file 1). Regarding foetal fluids/blood sera from foetuses, almost of all them were negative, except from two twins in G2, culled at 26 dpi (titres of 1:64 and 1:128), and two foetuses from G3, one aborted on day 12 pi (titre of 1:16) and the other culled at day 26 pi (titre 1:32) (Additional file 1). Specific IgG responses were not detected in control ewes or foetuses.

### Pathology and lesion quantification

#### Gross lesions

Partial detachment of the placenta was observed in those ewes that aborted and where the foetuses had not been expelled before at the day of culling (two in G1, day 12 pi, one in G2, day 12 pi and one in G3, day 19 pi). Aborted foetuses, either still in utero at the time of necropsy or already expelled, showed a variable degree of autolysis and friable viscera (Figure 2). The dead foetuses from G1 showed abdominal haemorrhage and subcutaneous oedema (Figure 3), while those from G2 to G3 showed a brownish coloured skin and a variable degree of mummification (Figure 3).

No macroscopic lesions were found in the cotyledons, or intercotyledonary membranes, of the placenta. However, placentas from dead foetuses, showed a variable degree of autolysis that, in several occasions, hampered proper examination. No macroscopic lesion was found in the maternal viscera, placentas or foetuses from ewes which had not aborted, either from challenged or control groups.

#### Microscopic lesions

##### Acute phase of the disease

In the three groups, and among those ewes with aborted foetuses between days 9 and 14 dpi, lesions consistent with ischemic necrosis (infarcts) were found in the placenta. This was characterized by large areas of congestion involving the whole depth of the placentome and neatly demarcated from the healthy tissue. The trophoblast cells showed necrosis, with an eosinophilic, homogeneous cytoplasma and fragmentation of the nuclear chromatin. The foetal mesenchyme within these areas was opaque, in contrast with its clear appearance in the non-affected areas. There was no evident infiltration of inflammatory cells in these areas. This type of lesion was found in the placenta from two ewes culled at day 12 dpi from G1, one sheep which aborted on day 13 pi in G2 and one ewe that aborted at day 9 pi from G3. It should be highlighted that most of the placentas from aborted foetuses were too autolytic to allow the identification of histological lesions.

In the brain of five of these foetuses (two in G2, at days 11 and 14 pi, and three in G3, at days 9 and 13 pi) areas of leukomalacia with no infiltration of inflammatory cells was found in the corona radiata and periventricular areas.

As these abortions occurred during the acute phase of the disease, and are considered to be caused by mechanisms different to those of the classical abortions in ovine toxoplasmosis [6] the lesions found in these placentas and foetuses were not considered for the quantification of histological lesions.

Data from the animals showing lesions non attributable to the acute phase of *T. gondii* infection are shown in Table 2, that contains the mean values of lesion size and number from placenta as well as foetal liver, lung and brain. Individual values for each foetus or placental sample studied, and showing lesions, are detailed in Additional file 2. Due to the few foetuses/placentas showing lesions, no statistical analysis was possible.

**Table 2:** **Average values of histological scoring in placenta and foetal viscera from each group**

Group	dpi	Placenta			Foetal brain			Foetal liver			Foetal lung		
		No. foci/cm^2^	ASF (mm^2^)	%LES	No. foci/cm^2^	ASF (mm^2^)	%LES	No. foci/cm^2^	ASF (mm^2^)	%LES	No. foci/cm^2^	ASF (mm^2^)	%LES
G1		–	–	–	–	–	–	–	–	–	–	–	–
G2		–	–	–	–	–	–	–	–	–	–	–	–
G3	19	–	–	–	–	–	–	0.58	0.024	0.01	1.65	0.01	0.01
G1	26	0.52	0.02	0.01	5.53	0.01	0.08	2.72	0.06	0.20	25.43	0.04	0.93
G2	26	0.91	0.05	0.05	12.96	0.03	0.31	8.71	0.05	0.58	16.10	0.06	0.90
G3	20–26^a^	na^b^	na	na	1.20	0.02	0.02	6.23	0.03	0.23	3.76	0.03	0.13

^a^Stillbirths occurred at 21, 22 and 26 dpi.

^b^The placenta from stillbirths was too autolytic to allow histological evaluation. dpi: days post infection when abortion occurred. ASF: Average size of focus.  %LES: Percentage of section affected by lesions.

##### Placenta

Placental lesions were only found in ewes culled at 26 dpi: one out of three in G1, carrying two dead foetuses, and two out of two in G2, one of these carrying one dead foetus and a viable one and the other ewe with two viable foetuses at the time of necropsy. It should be highlighted that most of the placentas from aborted foetuses or stillbirths were too autolytic to allow proper histological examination. Therefore, from G3 there were only three placentas available, two from day 12 pi and one from day 19 pi, where foetuses were viable and none of them showed histologic lesions.

**Figure 4:**
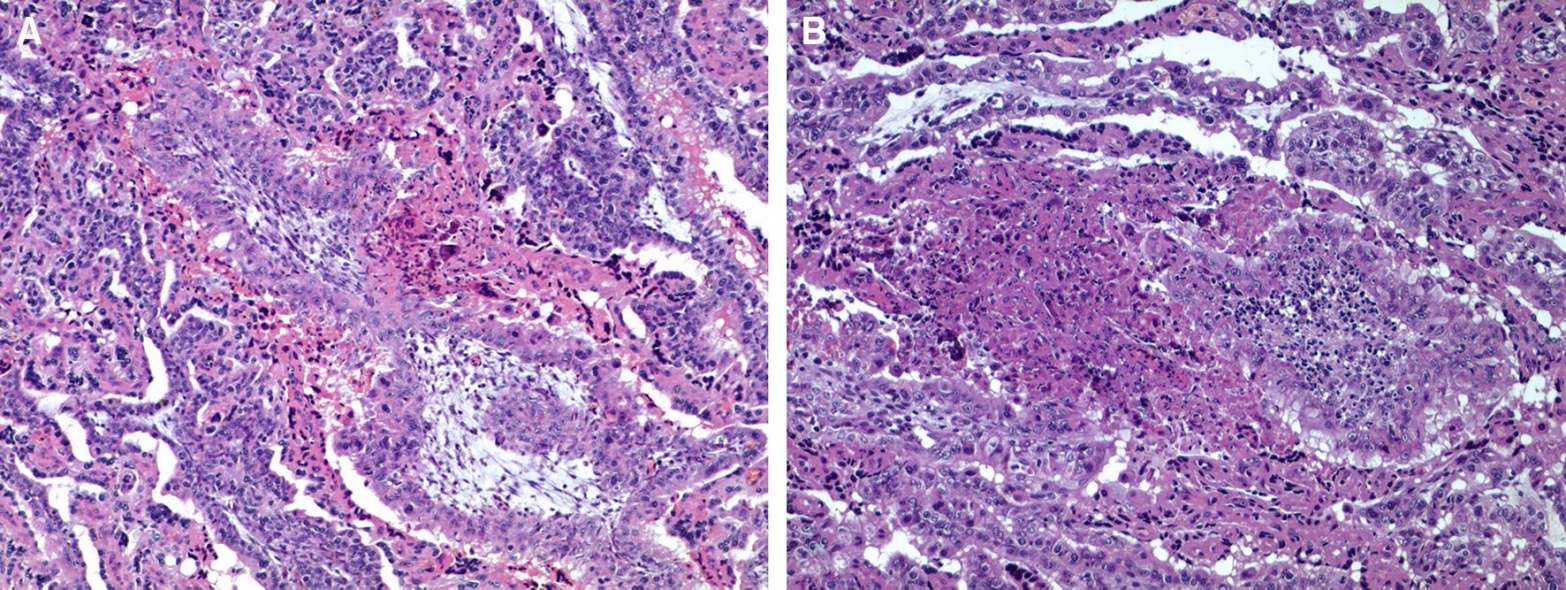
**Microscopic lesions in the placenta.** **A** Mild placentitis from a ewe challenged at 40 dg (G1). It is characterized by serum leakage between the maternal and foetal tissues, focal necrosis of foetal villi and non-purulent inflammation in the adjacent maternal septum. **B** Moderate to severe placentitis from a ewe challenged at 90 dg (G2). The lesion is denoted by large area of necrosis affecting both maternal and foetal villi and scarce infiltration of inflammatory cells.

The placenta from G1 showed small necrotic foci mainly located at the caruncular septa of the placentome. The foetal mesenchyma in the vicinity of these foci showed infiltration of mononuclear cells, morphologically consistent with lymphocytes and macrophages (Figure 4). In G2, one out of the two placentas with lesions showed larger foci of necrosis at the interdigitated area of the placentome, involving both maternal caruncular septa and adjacent foetal mesenchyme (Figure 4). There was local infiltration of mononuclear inflammatory cells, possible macrophages and lymphocytes, although it was not a prominent feature. The percentage of damaged area was eight time higher than in the placenta with lesions of G1. The lesions found in the other placenta from G2 were similar to those found in G1.

##### Foetal brain

**Figure 5:**
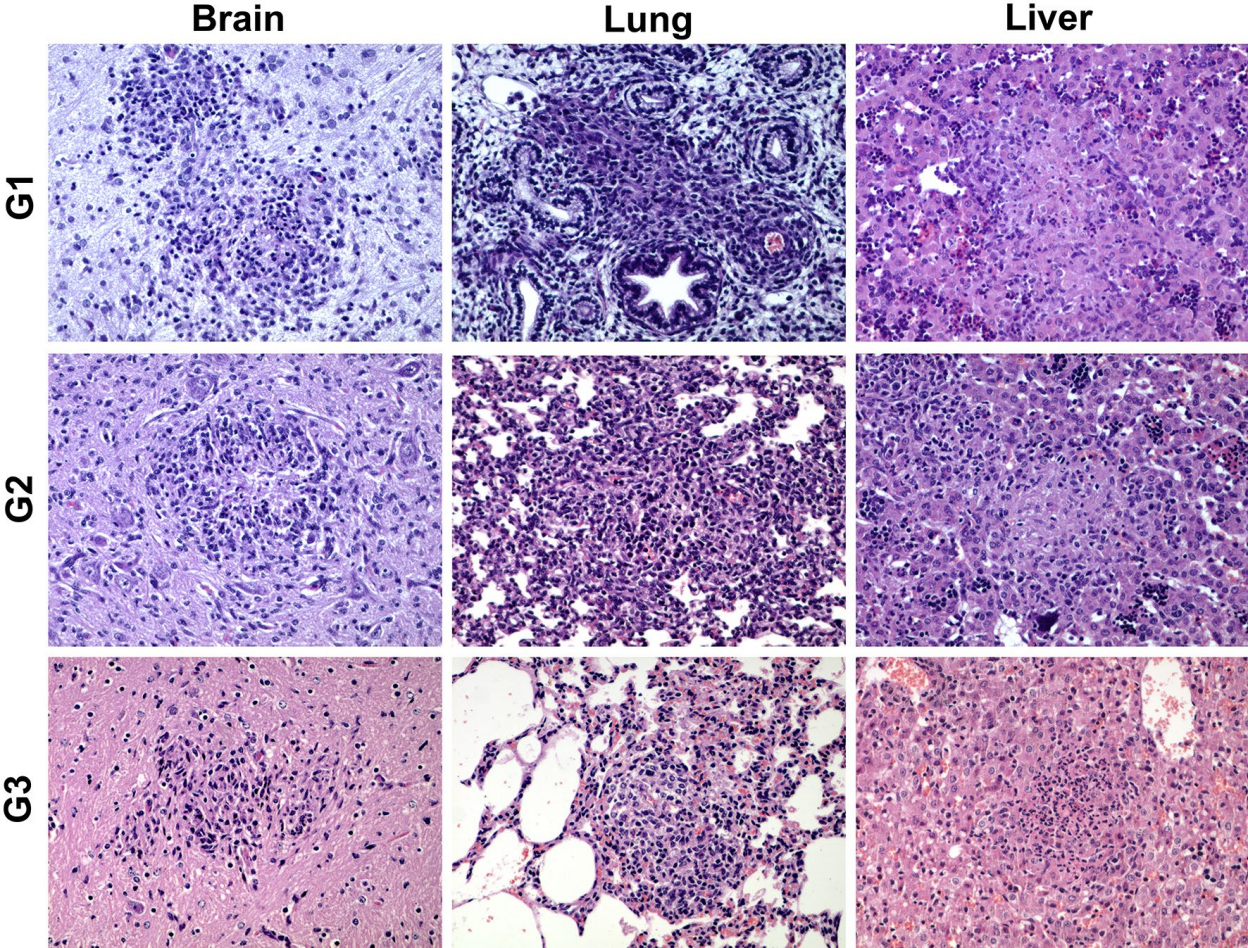
**Comparison of the characteristic microscopic lesions found in foetal brain, lung and liver at 20–26 dpi.** Pictures show the histological changes measured for the quantification of lesions. Both necrotic foci (more evident in liver of G1 and G2) and inflammatory cell aggregates (more evident in lung from G1) were measured.

Histological lesions in the brain were only observed at day 26 pi in G1 and G2 and from day 20 pi in G3. In the three groups, the lesions were characterized by the random distribution of mononuclear cells aggregates. While the cellular component of these foci was very similar among the three groups, only those from G2 showed central areas of necrosis. In G2, the foci were more numerous and slightly bigger (Figure 5), resulting in a higher percentage of damaged area when compared to G1 and G3 (Table 2).

##### Liver

Lesions in the liver were found earlier in G3, where they appear from day 19 pi, while in G1 and G2 they were only found at day 26 pi (Table 2). In G3 at day 19 pi this was a very mild perivascular infiltration of mononuclear cells. When comparing the lesions found from day 20 pi in the three groups, all of them were characterized by the presence of multiple foci of coagulative necrosis randomly distributed in the hepatic parenchyma. While in G1 the necrosis was the predominant feature of the foci, in G2 there was also infiltration of mononuclear inflammatory cells in the periphery of the foci and in G3 this infiltration became the main feature of the lesion (Figure 5). Regarding the number of foci and their size, there were no clear differences between groups.

##### Lung

Similarly to the liver, lesions in the lung were found earlier in G3, from day 19 pi, while only at day 26 pi in G1 and G2. In the only foetus showing lesions at 19 pi, there was a very mild perivascular infiltration of inflammatory cells. Lesions found from day 20 pi in the three groups were similar, mainly characterized by randomly distributed aggregates of non-purulent inflammatory cells (Figure 5). These aggregates were more numerous in G1 and G2 than in G3. No necrosis was found in relation to these foci in any of the groups.

No lesions were found in any of the non-challenged foetuses.

### Parasite distribution and burden in placental and foetal tissues

#### Placenta

##### *Toxoplasma*

**Figure 6:**
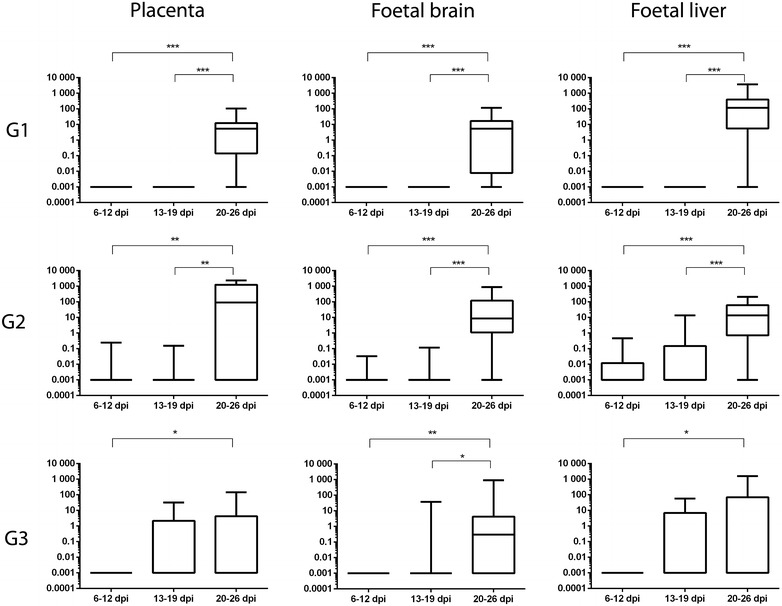
**Box-plot graph comparing** ***T. gondii*** **burdens measured at different dpi, within the same group, at the placenta and foetal viscera from the three groups.** Box-plot graphs represent the median burden, the lower and upper quartiles (boxes) and minimum and maximum values (whiskers). *P < 0.05, **P < 0.01 and ***P < 0.001 significant differences between dpi in each tissue.

DNA was detected in all samples from challenged animals, including aborted, stillbirths or culled ewes, studied between days 20 and 26 pi in the three groups. Between days 13 and 19 pi, it was detected only in G2 and G3 and in a lower percentage of cases than in the 20–26 dpi period. At the earliest period, between days 9 and 12 pi, *T. gondii* DNA was only detected in G2 and only in one third of the cases (Table 3). The mean parasite burden, measured as number of tachyzoites per mg of tissue, was significantly higher in days 20–26 pi, when compared to the previous periods (6–12 and 13–19 dpi) in G1 (*P* < 0.001) and G2 (*P* < 0.005), while in G3 this difference was only significant (*P* < 0.05) when compared with 6–12 dpi (Figure 6).

**Table 3:** **Percentages of cases showing histological lesions and parasite detection in placenta and foetal viscera from infected animals during the experiment**

		Placenta		Foetal brain		Foetal liver		Foetal lung	
Group	dpi	H/E^a^ (%)	PCR^b^ (%)	H/E (%)	PCR (%)	H/E (%)	PCR (%)	H/E (%)	PCR (%)
G1	6–12	–	–	–	–	–	–	–	16
	13–19	–	–	–	–	–	–	–	20
	20–26	33	100	28	86	28	100	86	100
G2	6–12	–	33	–	–	–	40	–	20
	13–19	–	50	–	18	–	36	–	45
	20–26	100	100	100	100	100	100	66	100
G3	6–12	–	–	–	–	–	–	–	–
	13–19	–	66	–	50	20	50	20	50
	20–26	na	100	33	80	33	60	66	80

^a^Histological lesions characteristic of *T. gondii* infection.

^b^Parasite detection by PCR amplification.

#### Foetal tissues

Regarding foetal tissues, the highest percentage of positive cases was observed in the brain, liver and lung of foetuses from G1 and G2 at the period of 20–26 dpi (Table 3), whereas, at the same period, the percentage of positive cases was lower in G3. Similarly as in placenta, in the period between days 13 and 19 pi these three organs were positive in G2 and G3, although at a lower percentage, while only 20% of cases from G1 showed parasite DNA in the lung. In the earliest period of sampling, between days 9 and 12 pi, only lung from G1 and G2, and also liver in the latter, showed detection of parasite DNA, although the rate of positive cases was not that different from those at 13–19 dpi in the same groups (Table 3). When comparing parasite burdens found in foetuses, similarly as observed in placentas, the burden in G1 and G2 was significantly higher (*P* < 0.001) in 20–26 dpi than in previous periods while in G3 the difference was less significant (*P* < 0.05 and *P* < 0.01) in brain and in liver it was only significant (*P* < 0.01) with the 6–12 dpi period (Figure 6).

When comparing the burden identified in the three organs (placenta, brain and liver) of the groups within the same period of time (Additional file 3), between 6 and 12 dpi, and in accordance with parasite detection (Table 3) the parasite burden was higher in G2 (*P* < 0.01 when compared with G1). Between days 13 and 19 pi, the burden was higher in G3, although the differences were only significant (*P* < 0.01) when compared with G1 in the placenta. Finally, between days 20 and 26 pi, when most of the samples were positive (Table 3), the burden found in G3 was lower (*P* < 0.01) in comparison to G2, in the placenta, or to G1 in the liver (Additional file 3). Individual data of parasite detection is shown in Additional file 4.

## Discussion

Despite the fact that the influence of pregnancy on the pathogenesis of ovine toxoplasmosis has been already highlighted [2, 12], the mechanisms responsible for this effect are yet unknown and there are few experimental studies that focused on this aspect of congenital toxoplasmosis, especially in sheep [1]. Recent studies in murine experimental models have shown that the gestation period when infection takes place has a direct effect on the consequences of the offspring but [13] due to several differences between mice and sheep in the histological architecture of placenta, immune response and duration of pregnancy, it is difficult to extrapolate results from the murine experimental model to sheep [14]. In order to provide insights into the pathogenesis of ovine toxoplasmosis, in this work an experimental study has been conducted for a detailed comparison of the influence of the stage of gestation on the pathogenesis of the disease. For this purpose, pregnant primiparous sheep with a similar genetic background (i.e. coming from the same flock) were challenged, at the three periods of gestation, with the same isolate, within the same experiment.

One striking finding in this experiment was the high number of abortions during the acute phase of the disease, in the first 2 weeks post infection, regardless of the stage of gestation when ewes were infected (i.e. they occurred in the three challenged groups). The occurrence of abortions in this phase of the disease has already been described in experimental studies but never with doses below 200 sporulated oocysts [4–6, 15, 16]. Comparing the abortion rate provoked by infection with 50 sporulated oocysts (i.e. 22.2%), with that found after infection with 2000 oocysts (91.6% abortion rate) and 500 oocysts (58.6% abortion rate) in a previous experiment [6], it is tempting to hypothesize that there is a correlation between the dose of infection and the rate of abortion during the acute phase of the disease. On the other hand, a dose of 200 oocysts was proposed to be the minimum infective dose for sheep [1]. A remarkable finding from this experiment is that a dose as low as 50 sporulated oocysts is capable to cause such a high rate of abortions in the acute phase of the disease throughout all gestation periods, and foetal pathology later on. Whether the isolate used in this experiment (i.e. M4) is more virulent or churra breed are more susceptible to *T. gondii* infection are two possible hypotheses that could explain such high rate of abortions and deserve further evaluation. The occurrence of abortions in the acute phase of the disease had been previously described after infections in mid and late gestation, 90 and 120 dg [4–6, 15, 16]. In the current study, they also occurred after infection at early gestation (day 40 of gestation), and at a similar rate to that in the other two groups. The pathogenesis of the abortions in the acute phase of ovine toxoplasmosis has been suggested to be different from that of the more classical abortions associated with the disease [4, 6]. The results from the present study, where very little parasite was detected in the placentomes or foetuses from these abortions, and where histological lesions were different to those classically described in ovine toxoplasmosis, confirm that suggestion. Bearing in mind the results from the present experiment, where no differences in the lesions or occurrence rate where observed between the three groups, it seems that these mechanisms, responsible for the abortion in the acute phase, are not influenced by the time of gestation when the ewes are challenged.

By contrast, there was a clear influence of the gestational stage over those abortions or stillbirths that occurred after 14 dpi. This influence is denoted by the differences in the rate and dpi of occurrence of abortions, histological lesions (scored depending on their number and size) and parasite burden,

While only one sheep per group was found to abort (i.e. carry a dead foetus) in G1 and G2, at 26 dpi in both, up to four sheep delivered stillbirths in G3 from 19 dpi. Previous studies where sheep or goats were challenged in late gestation also described the occurrence of abortions/stillbirths before the due date [3, 17, 18]. However, it has also been shown that, when gestation is allowed to continue after challenge, infection earlier in gestation results in more dead foetuses than during infections later in pregnancy [2]. Previous studies have shown that it takes at least 40 days for the abortion to occur after infection in mid pregnancy [15, 19], and this might be the reason why there were fewer abortion in G1 or G2 than G3, as the last culling day was 26 dpi. The fact that foetal death, which resulted in stillbirth, occurred earlier in G3 could be related to the earlier appearance of the lesions when sheep are infected at late gestation, since one foetus culled at day 19 pi, although still alive, already had lesions, while there were none in the foetuses culled from G1 or G2 until day 26 pi. This finding is in accordance with previous experimental infections at early or mid-gestation, where placental lesions were found after 28 dpi [20]. A different study reported placental lesions as soon as 10 dpi in ewes challenged at 60 or 90 dg but they were mild lesions, characterized by several foci of necrosis that become larger as the infection progressed. The earlier identification of lesions in this study might be related to the subcutaneous inoculation of tissue cysts as method of challenge, as opposed to oral infection with sporulated oocysts employed in majority of experimental challenges in sheep [1]. Differences in the distribution of the parasite and kinetics of the infection depending on the route of infection have been observed in cattle challenged with *N. caninum*, a closely related parasite to *T. gondii* [21].

Regarding the histological characteristics of these lesions, the presence of necrosis was the main histopathological change found in the placenta, liver and brain. This finding is in accordance with descriptions in previous experimental infections of sheep [2]. The pathogenesis of this type of lesion is not clear. While some authors have suggested, based on infection of genetically modified mice, that the participation of γ-interferon, secreted by T lymphocytes, is necessary for the necrosis of tissues after *T. gondii* infection [22]. Other experimental studies, also in genetically modified mice, have described the occurrence of necrosis in γ-interferon ^−^/^−^ mice [23]. Furthermore, it has been shown in cell culture of trophoblasts that the necrosis of these cells is not caused by the intracellular multiplication of the parasite nor by an immunomediated mechanism [24]. The strong correlation between parasite burden and necrosis, and the limited infiltration of inflammatory cells in these lesions suggests that the presence of the parasite by itself is the trigger signal for the lesion through a non-immune mediated mechanism, or at least, not only immune mediated. Furthermore, necrotic lesions were more prominent in G2 and, to a lesser extent in G1, where the burden of parasite was higher than in G3, adding to the hypothesis that the main factor involved in the development of necrosis is the presence and multiplication of the parasite. The maturation of the foetal immune response in G3 would allow the foetus to control parasite multiplication [2], hence the lower burdens and the milder necrotic lesions. On the other hand, studies based on cell culture of trophoblasts have suggested that local factors, secreted by the infected trophoblasts, could cause the necrosis of adjacent cells through a bystander effect [24]. The relation between the parasite and the release of local factors and whether it occurs also in other organs, such as liver or brain, deserves further investigation.

It was interesting to find that, while lesions in placenta, liver and, to a lesser extent, brain were characterized by the presence of necrosis with or without inflammation, in the lung there was no necrosis and only perivascular aggregation of inflammatory cells was found, mainly in G1 and G2. Although multifocal necrosis in the foetal lung have been found after infection at early and mid gestation [2], other experimental studies, in sheep or goats, have reported predominantly inflammatory lesions in the lung and only minimal necrosis [3, 25]. The reason why necrosis, a common finding associated with foetal toxoplasmosis, is not so frequent in this organ is not clear and the further research of its pathogenesis could contribute to the better understanding of tissue damage associated with *T. gondii* infection.

When comparing the severity of foetal lesions at 26 dpi between the three groups, those more severe are found in G2, when ewes were challenged at mid gestation. Previous experimental infections also described that lambs/stillbirths from ewes challenged at late gestation showed milder lesion than foetuses from ewes challenged at mid gestation [3]. The strong effect of the day of gestation over the development of lesions is shown by the fact that infection at day 60 of gestation caused more severe lesions than infection at day 40 or 90 of gestation, as had been observed in the present experiment but also in previous studies [2].

There was a close relation between the presence of pathological changes found in the placenta or foetus and the quantity of parasite measured in at the same locations. Thus, it seems that there is a narrow window of time, around day 60 and 90 of gestation, when lesions are more severe and this fact might be related with a higher parasite burden. Molecular amplification of parasite DNA allows the detection of *T. gondii* in tissues as soon as 12 dpi in G2. These results confirm that parasite could reach the placenta and foetus by day 10 pi [2], and the presence of the parasite is necessary for the development of histological lesions. Despite the amplification of parasite DNA in the limited number of available samples, the parasite was highly detected (in more than 50% of the samples) at 26 dpi in G1, 19 dpi in the placenta of G2 and 19 dpi in the placenta and foetus from G3. Similar results are found when analysing parasite burden, where it can be seen that, at 19 dpi, higher burdens are found in G3 when comparing it to G1 and G2. This finding may suggest that the parasite is able to either reach sooner or multiply easily in the foetus and placenta of G3 than in G1 and G2. When comparing the burdens and percentage of detection during all time points within the same group, both G1 and G2 showed a sharp increase at 26 dpi. The same tendency has also been described in a previous experimental infection at mid gestation [20]. The same tendency in parasite burden and percentage of detection is also found in G3 but the differences with 12 and 19 dpi are less significant. Actually, when comparing these parameters at 26 dpi between the three groups, G3 showed the lowest parasite burden. It could be tempting to hypothesize that despite being found in the placenta and foetus sooner in G3, the parasite does not multiple so efficiently afterwards, possibly due to a more efficient foetal immune response, but it is not efficient enough to protect from foetal death.

The explanation for this finding is yet unclear, as there are no previous studies comparing the burden of parasite in placenta or foetuses at the three trimesters of gestation or particularly studying the kinetic of parasite multiplication in late pregnancy. The maturation of the foetal immune system, and therefore its ability to respond against the parasite has been suggested as the main factor involved in the milder effects of the infection at late pregnancy [2]. A plausible explanation for the lower burden and percentage of detection at 26 dpi in G3, when compared to G1 and G2, could be then that the foetal immune system, at late pregnancy, is able to control the parasite multiplication while at early and mid-gestation it might not be as efficient. This may also explain why lesions in G3 were milder than in the other two groups. The lower burden of parasite in G3 possibly induced milder lesions. Another indication of this difference in immune system maturation among groups was that IFAT analysis of foetal sera or fluids only showed positive titres at 26 dpi in G2 and G3, but not in G1.This result, were no antibodies were detected before 26 dpi is in accordance with previous studies showing the absence of *T. gondii* specific IFAT titres before 30 dpi [2] and, tangentially, shows that this technique could suffer from little sensitivity when diagnosing natural cases of abortions, as only one stillbirth, out of the nine aborted foetuses/stillbirths observed had a positive titre.

A most interesting finding was that the parasite reached the placenta and foetus earlier in G3 than in G1 and G2, suggesting that there is a deficient control of its dissemination at late gestation when compared to early and mid-pregnancy. A shift in the maternal immune response, from Th1 towards a Th2 cytokine microenvironment, during pregnancy has been proposed as a key element to sustain pregnancy in murine experimental models in what was coined the Th1/Th2 paradigm [26]. Bearing in mind that a Th1 response is key in the control of *T. gondii* infection [27], this paradigm could be the explanation why the parasite reached the placenta and foetus earlier, or multiplied faster, in those ewes challenged at late gestation. However, this paradigm has been regarded as an over simplification of the materno-foetal relationship [28, 29], based on studies in murine experimental models, which makes it difficult to extrapolate results to outbreed species [30]. Recent studies have failed to find variations in the peripheral immune response during ovine gestation, suggesting that if immune modulation occurs in sheep, it would act at a local level [31]. Regarding the importance of local factors during pregnancy, the study of natural killer cells is gaining increasing relevance for the role in maintaining pregnancy [28, 32]. It is interesting to notice that, in the present study, ewes challenged at early gestation showed a longer period of fever compared to those challenged at mid and late gestation but also the latest detection of the parasite. Bearing in mind that the induction of fever is a consequence of the innate immune system and has been shown to boost the effectiveness of the adaptive immune response [33], it is tempting to hypothesize that the differences found between G1 and G3 might not only be linked with a possible Th1/Th2 shift, but also with variations in the innate immune response.

Overall, the results from the present experiment confirm, and substantiate the clear influence of the time of gestation when sheep are challenged on the pathogenesis of ovine toxoplasmosis, adding information to previous studies [2]. Its influence was clearly shown in the clinical development of the disease, as well as in the severity of lesions and parasite burden. Parasite dissemination was better controlled in early than mid gestation, therefore causing fewer lesions. Infection in the last third of gestation was associated with a faster dissemination of the parasite to foetus and placenta causing the occurrence of abortion. However, lesions in the foetuses were less severe than those caused by infection earlier in pregnancy. The mechanisms responsible for these variation are yet unknown, although it has been widely suggested that a modulation of the immune response can occur during pregnancy [28, 30]. The type of modulation and its influence upon peripheral and local immune responses, as well as the importance of maternal innate immune response, deserve further experimental evaluation.

## **Additional files**

10.1186/s13567-016-0327-z **Individual serological titres in infected dams and foetuses/stillbirths at the time of necropsy**. Table showing the individual serological titres in infected dams and foetuses/stillbirths at the time of necropsy.
10.1186/s13567-016-0327-z **Individual quantification of lesions in the placenta, foetal liver, lung and brain**. Table showing the individual quantification of lesions in the placenta, foetal liver, lung and brain.
10.1186/s13567-016-0327-z **Box-plot graph** ***T. gondii*** **burdens measured at the same period post infection and comparing between the three groups at the placenta and foetal viscera**. Box-plot graphs represent the median burden, the lower and upper quartiles (boxes) and minimum and maximum values (whiskers). (*) indicates *P* < 0.05 significant differences between groups in each period post infection.
10.1186/s13567-016-0327-z **Individual frequency of parasite DNA detection in infected animals**. Table showing the individual frequency of parasite DNA detection in infected animals.

## References

[1] Dubey JP (2009). Toxoplasmosis of animals and humans.

[2] Buxton D, Finlayson J (1986). Experimental infection of pregnant sheep with *Toxoplasma gondii*: pathological and immunological observations on the placenta and foetus. J Comp Pathol.

[3] Buxton D, Gilmour JS, Angus KW, Blewett DA, Miller JK (1982). Perinatal changes in lambs infected with *Toxoplasma gondii*. Res Vet Sci.

[4] Owen MR, Clarkson MJ, Trees AJ (1998). Acute phase toxoplasma abortions in sheep. Vet Rec.

[5] Trees AJ, Crozier SJ, Buxton D, Blewett DA (1989). Serodiagnosis of ovine toxoplasmosis: an assessment of the latex agglutination test and the value of IgM specific titres after experimental oocyst-induced infections. Res Vet Sci.

[6] Castaño P, Fuertes M, Ferre I, Fernández M, Ferreras Mdel C, Moreno-Gonzalo J, González-Lanza C, Katzer F, Regidor-Cerrillo J, Ortega-Mora LM, Pérez V, Benavides J (2014). Placental thrombosis in acute phase abortions during experimental *Toxoplasma gondii* infection in sheep. Vet Res.

[7] Buxton D (1990). Ovine toxoplasmosis: a review. J R Soc Med.

[8] Arranz-Solis D, Benavides J, Regidor-Cerrillo J, Fuertes M, Ferre I, Ferreras Mdel C, Collantes-Fernandez E, Hemphill A, Perez V, Ortega-Mora LM (2015). Influence of the gestational stage on the clinical course, lesional development and parasite distribution in experimental ovine neosporosis. Vet Res.

[9] Gutierrez J, O’Donovan J, Proctor A, Brady C, Marques PX, Worrall S, Nally JE, McElroy M, Bassett H, Fagan J, Maley S, Buxton D, Sammin D, Markey BK (2012). Application of quantitative real-time polymerase chain reaction for the diagnosis of toxoplasmosis and enzootic abortion of ewes. J Vet Diagn Invest.

[10] Alvarez-García G, Collantes-Fernández E, Costas E, Rebordosa X, Ortega-Mora LM (2003). Influence of age and purpose for testing on the cut-off selection of serological methods in bovine neosporosis. Vet Res.

[11] Hurtado A, Aduriz G, Moreno B, Barandika J, Garcia-Perez AL (2001). Single tube nested PCR for the detection of *Toxoplasma gondii* in fetal tissues from naturally aborted ewes. Vet Parasitol.

[12] Innes EA, Bartley PM, Buxton D, Katzer F (2009). Ovine toxoplasmosis. Parasitology.

[13] Wang Y, Wang M, Wang G, Pang A, Fu B, Yin H, Zhang D (2011). Increased survival time in mice vaccinated with a branched lysine multiple antigenic peptide containing B- and T-cell epitopes from *T. gondii* antigens. Vaccine.

[14] Innes EA (1997). Toxoplasmosis: comparative species susceptibility and host immune response. Comp Immunol Microbiol Infect Dis.

[15] Buxton D, Thomson KM, Maley S (1993). Treatment of ovine toxoplasmosis with a combination of sulphamezathine and pyrimethamine. Vet Rec.

[16] Buxton D, Brebner J, Wright S, Maley SW, Thomson KM, Millard K (1996). Decoquinate and the control of experimental ovine toxoplasmosis. Vet Rec.

[17] Dubey JP (1981). Toxoplasma-induced abortion in dairy goats. J Am Vet Med Assoc.

[18] Kirkbride CA, Dubey JP, Libal MC (1992). Effect of feeding lasalocid to pregnant ewes experimentally infected with *Toxoplasma gondii*. Vet Parasitol.

[19] Engeland IV, Waldeland H, Kindahl H, Ropstad E, Andresen O (1996). Effect of *Toxoplasma gondii* infection on the development of pregnancy and on endocrine foetal-placental function in the goat. Vet Parasitol.

[20] Gutierrez J, O’Donovan J, Williams E, Proctor A, Brady C, Marques PX, Worrall S, Nally JE, McElroy M, Bassett H, Sammin D, Buxton D, Maley S, Markey BK (2010). Detection and quantification of *Toxoplasma gondii* in ovine maternal and foetal tissues from experimentally infected pregnant ewes using real-time PCR. Vet Parasitol.

[21] Benavides J, Collantes-Fernandez E, Ferre I, Perez V, Campero C, Mota R, Innes E, Ortega-Mora LM (2014). Experimental ruminant models for bovine neosporosis: what is known and what is needed. Parasitology.

[22] Liesenfeld O, Kosek J, Remington JS, Suzuki Y (1996). Association of CD4 + T cell-dependent, interferon-gamma-mediated necrosis of the small intestine with genetic susceptibility of mice to peroral infection with *Toxoplasma gondii*. J Exp Med.

[23] Silva NM, Vieira JC, Carneiro CM, Tafuri WL (2009). *Toxoplasma gondii*: the role of IFN-gamma, TNFRp55 and iNOS in inflammatory changes during infection. Exp Parasitol.

[24] Abbasi M, Kowalewska-Grochowska K, Bahar MA, Kilani RT, Winkler-Lowen B, Guilbert LJ (2003). Infection of placental trophoblasts by *Toxoplasma gondii*. J Infect Dis.

[25] Dubey JP (1988). Lesions in transplacentally induced toxoplasmosis in goats. Am J Vet Res.

[26] Wegmann TG, Lin H, Guilbert L, Mosmann TR (1993). Bidirectional cytokine interactions in the maternal-fetal relationship: is successful pregnancy a TH2 phenomenon?. Immunol Today.

[27] Tan F, Hu X, Luo FJ, Pan CW, Chen XG (2011). Induction of protective Th1 immune responses in mice by vaccination with recombinant *Toxoplasma gondii* nucleoside triphosphate hydrolase-II. Vaccine.

[28] Chaouat G, Ledee-Bataille N, Dubanchet S, Zourbas S, Sandra O, Martal J (2004). Reproductive immunology 2003: reassessing the Th1/Th2 paradigm?. Immunol Lett.

[29] Zenclussen AC (2005). CD4(+)CD25 + T regulatory cells in murine pregnancy. J Reprod Immunol.

[30] Entrican G (2002). Immune regulation during pregnancy and host-pathogen interactions in infectious abortion. J Comp Pathol.

[31] Wattegedera S, Rocchi M, Sales J, Howard CJ, Hope JC, Entrican G (2008). Antigen-specific peripheral immune responses are unaltered during normal pregnancy in sheep. J Reprod Immunol.

[32] Jabrane-Ferrat N, Siewiera J (2014). The up side of decidual natural killer cells: new developments in immunology of pregnancy. Immunology.

[33] Evans SS, Repasky EA, Fisher DT (2015). Fever and the thermal regulation of immunity: the immune system feels the heat. Nat Rev Immunol.

