# Supplementary material for: Experimental ovine toxoplasmosis: influence of the gestational stage on the clinical course, lesion development and parasite distribution
Source: Vet Res. 2016 Mar 16;47:43. doi: 10.1186/s13567-016-0327-z (PMC4793618; doi:10.1186/s13567-016-0327-z)
Supplement: Supplementary file 1 — 10.1186/s13567-016-0327-z Individual serological titres in infected dams and foetuses/stillbirths at the time of necropsy. Table showing the individual serological titres in infected dams and foetuses/stillbirths at the time of necropsy. [file 13567_2016_327_MOESM1_ESM.docx]

**Additional file 1 Individual serological titres in infected dams and foetuses/stillbirths at the time of necropsy.**

| **Group** | **Ewe ref.** | **Time of necropsy (dpi)** | **Dams sera titre** | **Foetus/Lambs ref.** | **FL or Sera titre** |
| --- | --- | --- | --- | --- | --- |
| **G1**  **(day 40)** | **146** | 12 | 1:400 | 146F1 | - |
|  |  |  |  | 146F2 | - |
|  | **147** | 12 | 1:800 | 147F1 | - |
|  |  |  |  | 147F2 | - |
|  | **148** | 12 | 1:800 | 148F1 | - |
|  |  |  |  | 148F2 | - |
|  | **150** | 19 | 1:1600 | 150F1 | - |
|  |  |  |  | 150F2 | - |
|  | **151** | 19 | 1:400 | 151F1 | - |
|  |  |  |  | 151F2 | - |
|  | **152** | 19 | 1:800 | 152F1 | - |
|  | **154** | 26 | 1:800 | 154F1 | - |
|  |  |  |  | 154F2 | - |
|  |  |  |  | 154F3 | - |
|  | **155** | 26 | 1:600 | 155F1 | - |
|  |  |  |  | 155F2 | - |
|  | **156** | 26 | 1:1600 | 156F1 | - |
|  |  |  |  | 156F2 | - |
| **G2**  **(day 90)** | **387** | 11^ | 1:400 | 387F1 | -* |
|  | **158** | 12 | 1:800 | 158F1 | -* |
|  | **159** | 12^ | 1:3200 | 159F1 | -* |
|  |  |  |  | 159F2 | -* |
|  |  |  |  | 159F3 | -* |
|  | **160** | 13^ | 1:3200 | 160F1 | - |
|  |  |  |  | 160F2 | - |
|  |  |  |  | 160F3 | - |
|  | **379** | 14^ | 1:3200 | 379F1 | - |
|  | **162** | 19 | 1:1600 | 162F1 | - |
|  |  |  |  | 162F2 | - |
|  | **163** | 19 | 1:3200 | 163F1 | - |
|  |  |  |  | 163F2 | - |
|  |  |  |  | 163F3 | - |
|  | **165** | 26 | 1:6400 | 165F1 | - |
|  |  |  |  | 165F2 | - |
|  | **166** | 26 | 1:6400 | 166F1 | 1:64 |
|  |  |  |  | 166F2 | 1:128 |
| **G3**  **(day 120)** | **179** | 9^ | 1:1600 | 179F1 | na |
|  | **181** | 12 | 1:1600 | 181F1 | 1:16 |
|  | **182** | 12 | 1:1600 | 182F1 | - |
|  | **183** | 13^ | 1:1600 | 183F1 | -* |
|  |  |  |  | 183F2 | -* |
|  | **185** | 19^ | 1:1600 | 185F1 | -* |
|  | **186** | 19 | 1:6400 | 186F1 | - |
|  | **187** | 21^ | 1:6400 | 187F1 | -* |
|  | **188** | 22^ | 1:3200 | 188F1 | -* |
|  |  |  |  | 188F2 | -* |
|  | **189** | 26^ | 1:12 800 | 189F1 | na |
|  |  |  |  | 189F2 | 1:32 |

^ aborted foetus or stillbirth. ^*^ analysis of thoracic sera from aborted foetuses. dpi: days post-infection; dg: days of gestation; NA: not available.
