# Supplementary material for: Experimental ovine toxoplasmosis: influence of the gestational stage on the clinical course, lesion development and parasite distribution
Source: Vet Res. 2016 Mar 16;47:43. doi: 10.1186/s13567-016-0327-z (PMC4793618; doi:10.1186/s13567-016-0327-z)
Supplement: Supplementary file 2 — 10.1186/s13567-016-0327-z Individual quantification of lesions in the placenta, foetal liver, lung and brain. Table showing the individual quantification of lesions in the placenta, foetal liver, lung and brain. [file 13567_2016_327_MOESM2_ESM.docx]

**Additional file 2 Individual quantification of lesions in the placenta, foetal liver, lung and brain**.

| **Group** | **Foetus reference** | **Time of necropsy** |  | **Placentome** | | |  | **Foetal liver** | | |  | **Foetal brain** | | |  | **Foetal lung** | | |
| --- | --- | --- | --- | --- | --- | --- | --- | --- | --- | --- | --- | --- | --- | --- | --- | --- | --- | --- |
|  |  |  |  | No. foci per cm^2^ | ASF (mm^2^) | %LES |  | No. foci per cm^2^ | ASF (mm^2^) | %LES |  | No. foci per cm^2^ | ASF (mm^2^) | %LES |  | No. foci per cm^2^ | ASF (mm^2^) | %LES |
| **G1**  **(day 40)** | **154F1** | 26 |  | - | - | - |  | - | - | - |  | - | - | - |  | 4,16 | 0,018 | 0,08 |
|  | **154F2** | 26 |  | - | - | - |  | - | - | - |  | - | - | - |  | 1,19 | 0,058 | 0.07 |
|  | **155F1** | 26 |  | 0.52 | 0.018 | 0.01 |  | 1.98 | 0.023 | 0.05 |  | 6.60 | 0.016 | 0.11 |  | 61.42 | 0.028 | 1.73 |
|  | **155F2** | 26 |  |  |  |  |  | - | - | - |  | 4.47 | 0.01 | 0.05 |  | 23.10 | 0.017 | 0.38 |
|  | **156F1** | 26 |  | - | - | - |  | - | - | - |  | - | - | - |  | 41.06 | 0.062 | 2.54 |
|  | **156F2** | 26 |  | - | - | - |  | 3.47 | 0.1 | 0.35 |  | - | - | - |  | 21.68 | 0.037 | 0.8 |
| **G2**  **(day 90)** | **165F1** | 26 |  | 0.88 | 0.021 | 0.02 |  | 14.39 | 0.076 | 1.09 |  | 3.66 | 0.039 | 0.14 |  | 13.88 | 0.053 | 0.74 |
|  | **166F1** | 26 |  | 0.08 | 0.95 | 0.085 |  | 3.06 | 0.025 | 0.08 |  | 7.46 | 0.033 | 0.24 |  | 1.83 | 0.026 | 0.05 |
|  | **166F2** | 26 |  |  |  |  |  | 2.99 | 0.018 | 0.06 |  | 37.07 | 0.019 | 0.69 |  | - | - | - |
| **G3**  **(day 120)** | **186F1** | 19 |  | - | - | - |  | 0.58 | 0.024 | 0.01 |  | - | - | - |  | 1.65 | 0.008 | 0.01 |
|  | **187F1** | 21^a^ |  | NA | NA | NA |  | 4.19 | 0.031 | 0.13 |  | - | - | - |  | - | - | - |
|  | **188F2** | 22^a^ |  | NA | NA | NA |  | 10.38 | 0.045 | 0.47 |  | - | - | - |  | 1.83 | 0.026 | 0.05 |
|  | **189F2** | 26^a^ |  | NA | NA | NA |  | 4.14 | 0.025 | 0.1 |  | 1.20 | 0.017 | 0.02 |  | 5.69 | 0.037 | 0.21 |

^a^ Stillbirths. The other cases were culled as per initial experimental design. dpi: days post-infection; dg: days of gestation; NA: not available. ASF: average size of lesional foci.
