# Supplementary figures and images for: Experimental ovine toxoplasmosis: influence of the gestational stage on the clinical course, lesion development and parasite distribution
Source: Vet Res. 2016 Mar 16;47:43. doi: 10.1186/s13567-016-0327-z (PMC4793618; doi:10.1186/s13567-016-0327-z)

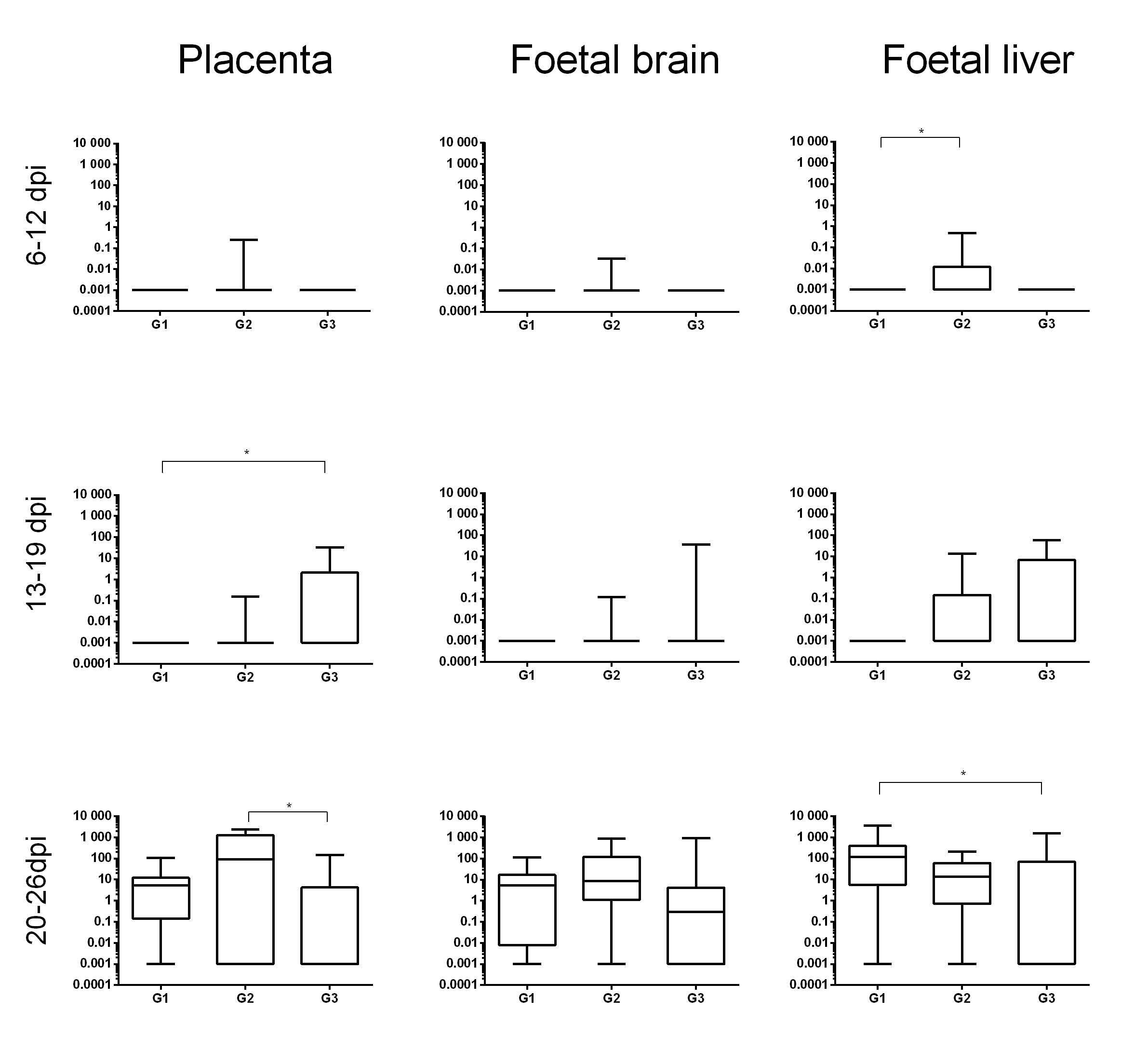

Supplement: Supplementary file 3 — 10.1186/s13567-016-0327-z Box-plot graph T. gondii burdens measured at the same period post infection and comparing between the three groups at the placenta and foetal viscera. Box-plot graphs represent the median burden, the lower and upper quartiles (boxes) and minimum and maximum values (whiskers). (*) indicates P < 0.05 significant differences between groups in each period post infection. [file 13567_2016_327_MOESM3_ESM.jpg]
