# Supplementary material for: Experimental ovine toxoplasmosis: influence of the gestational stage on the clinical course, lesion development and parasite distribution
Source: Vet Res. 2016 Mar 16;47:43. doi: 10.1186/s13567-016-0327-z (PMC4793618; doi:10.1186/s13567-016-0327-z)
Supplement: Supplementary file 4 — 10.1186/s13567-016-0327-z Individual frequency of parasite DNA detection in infected animals. Table showing the individual frequency of parasite DNA detection in infected animals. [file 13567_2016_327_MOESM4_ESM.docx]

**Additional file 4 Individual frequency of parasite DNA detection** **in infected animals.**

| **Group** | **Foetus reference** | **Time of necropsy** |  | **Placenta** |  | **Foetal tissues** | | | | |
| --- | --- | --- | --- | --- | --- | --- | --- | --- | --- | --- |
|  |  |  |  |  |  | Brain | Liver | Heart | Lung | STM |
| **G1**  **(day 40)** | **146F1** | 12 |  | - |  | - | - | - | + | + |
|  | **146F2** | 12 |  |  |  | - | - | - | - | - |
|  | **147F1** | 12 |  | - |  | - | - | - | - | - |
|  | **147F2** |  |  |  |  | - | - | - | - | - |
|  | **148F1** | 12 |  | - |  | -^ | - | - | - | - |
|  | **148F2** |  |  |  |  | - | - | - | - | - |
|  | **150F1** | 19 |  | - |  | -^ | - | - | -^ | - |
|  | **150F2** |  |  |  |  | -^ | -^ | - | +^ | - |
|  | **151F1** | 19 |  | - |  | - | - | - | - | - |
|  | **151F2** |  |  |  |  | - | - | - | - | - |
|  | **152F1** | 19 |  | - |  | - | - | - | - | - |
|  | **154F1** | 26 |  | +++ |  | +++ | +++ | +++ | +++ | +++ |
|  | **154F2** |  |  |  |  | -^ | ++ | +++ | +++ | +++ |
|  | **154F3** |  |  |  |  | +++ | +++ | +++ | +++ | +++ |
|  | **155F1** | 26 |  | +++ |  | +++ | +++ | +++ | +++ | +++ |
|  | **155F2** |  |  |  |  | +++ | +++ | +++ | +++ | +++ |
|  | **156F1** | 26 |  | +++ |  | ++ | +++ | +++ | +++ | +++ |
|  | **156F2** |  |  |  |  | +++ | ++ | +++ | +++ | +++ |
| **G2**  **(day 90)** | **387F1** | 11 |  | - |  | - ^ | - ^ | - ^ | - ^ | - ^ |
|  | **158F1** | 12 |  | + |  | ++ | + | + | - | - |
|  | **159F1** | 12 |  | - |  | -^ | ++ | - | - | - |
|  | **159F2** |  |  |  |  | -^ | - | - | - | - |
|  | **159F3** |  |  |  |  | -^ | - | - | - | - |
|  | **160F1** | 13 |  | + |  | - | - | - | - | - |
|  | **160F2** |  |  |  |  | - | - | - | - | - |
|  | **160F3** |  |  |  |  | + | - | - | +++ | - |
|  | **379F1** | 14 |  |  |  | - ^b^ | - ^b^ | - ^b^ | - ^b^ | - ^b^ |
|  | **162F1** | 19 |  | ++ |  | +++ | + | ++ | +++ | - |
|  | **162F2** |  |  |  |  | - | - | - | - | - |
|  | **163F1** | 19 |  | - |  | - | ++ | ++ | - | - |
|  | **163F2** |  |  |  |  | - | ++ | - | - | - |
|  | **163F3** |  |  |  |  | - | ++ | - | +++ | +++ |
|  | **165F1** | 26 |  | ++ |  | ++ | +++ | +++ | +++ | +++ |
|  | **165F2** |  |  |  |  | +++^ | ++ ^ | - | - | - |
|  | **166F1** | 26 |  | +++ |  | +++ | +++ | +++ | +++ | +++ |
|  | **166F2** |  |  |  |  | +++ | +++ | +++ | +++ | +++ |
| **G3**  **(day 120)** | **179F1** | 9 |  | - |  | - | - | - | - | - |
|  | **181F1** | 12 |  | - |  | - | - | - | - | - |
|  | **182F1** | 12 |  | - |  | - | - | - | - | - |
|  | **183F1** | 13 |  | - |  | - | - | - | - | - |
|  | **183F2** |  |  |  |  | - | - | - | - | - |
|  | **184F1** | 19 |  | - |  | - | - | - | - | - |
|  | **185F1** | 19 |  | +++ |  | + | - | +++ | +++ | +++ |
|  | **186F1** | 19 |  | ++ |  | + | +++ | +++ | +++ | +++ |
|  | **187F1** | 21 |  | + ^ |  | ++ | +++ | +++ | +++ | +++ |
|  | **188F1** | 22 |  | ++ ^ |  | ++ ^ | - ^ | +++ ^ | +++ ^ | +++^ |
|  | **188F2** |  |  |  |  | +++ | ++ | +++ | +++ | +++ |
|  | **189F1** | 26 |  | +++ ^ |  | - ^ | - ^ | - ^ | - ^ | -^ |
|  | **189F2** |  |  |  |  | +++ | ++ | +++ | +++ | +++ |

^ autolytic sample. dpi: days post-infection; dg: days of gestation; PLC: placenta; MLN: mesenteric lymph node; ILN: ileophemoral lymph node; STM semitendinosus muscle; na: sample not; plus (+++, ++, +) and minus (-) signs represent PCR detection in >67%, 66-34%, <33% and 0% of samples analysed, respectively.
